# Interferon-γ and IL-27 positively regulate type 1 regulatory T cell development during adaptive tolerance

**DOI:** 10.1016/j.isci.2025.112308

**Published:** 2025-03-28

**Authors:** David A.J. Lecky, Lozan Sheriff, Sophie T. Rouvray, Lorna S. George, Alastair Copland, Rebecca A. Drummond, David C. Wraith, David Bending

**Affiliations:** 1 Department of Immunology and Immunotherapy, School of Infection, Inflammation and Immunology, College of Medicine and Health, University of Birmingham, Birmingham B15 2TT, UK

**Keywords:** Immunology, Cell biology, Stem cell research

## Abstract

Strong T cell receptor (TCR) and interleukin (IL)-27 signaling influence type 1 regulatory (Tr1) T cell development, but whether other signals determine their differentiation is unclear. Utilizing Tg4 TCR transgenic mice, we established a model for rapid Tr1 cell induction. A single high dose of [4Y]-MBP peptide drove the differentiation of *Il10*^+^ T cells with Tr1 cell mRNA and protein signatures. Kinetic transcriptional and phenotypic analyses revealed that the Tr1 cell module was transient and preceded by *Ifng* transcription in other CD4^+^ T cells. Changes in Tr1 cell frequency correlated with altered macrophage activation, while neutralization of interferon (IFN)γ reduced Tr1 cell frequency and the TCR signal strength markers Nur77, inducible T cell costimulator (ICOS), and OX40. Antibody depletion experiments inferred that the relevant source of IFNγ was not natural killer (NK) cell derived. Additionally, blocking IL-27 in combination with IFNγ neutralization additively reduced Tr1 cell frequency *in vivo*. These findings reveal that IFNγ has a non-redundant role in augmenting Tr1 cell differentiation *in vivo*.

## Highlights

• A single high dose of modified self-peptide induces type 1 regulatory T cells (Tr1)
• Tr1 cell development is positively regulated by IFNγ
• Anti-IFNγ treatment reduces markers of strong TCR signaling and Tr1 cell frequency
• Blocking IL-27 in combination with anti-IFNγ additively reduces Tr1 cell frequency

## Graphical abstract

**Figure:**
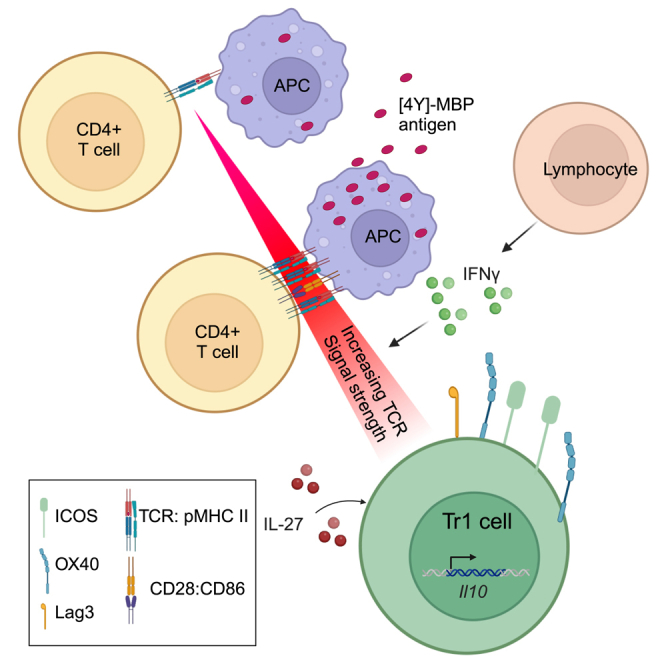

## Introduction

Interleukin (IL)-10 is an immunoregulatory cytokine produced by multiple immune cell types and has a key function in the regulation of adaptive immunity.^1^ IL-10 can inhibit antigen presentation, cell proliferation, and pro-inflammatory cytokines,^2^^,^^3^ including directly countering interferon (IFN)γ through inhibition of IFNγ-induced genes, including major histocompatibility complex (MHC) class II.^4^^,^^5^ MHC class II presents peptide to T cell receptor (TCR) complexes on CD4^+^ T cells to promote T cell activation. IL-10 inhibits antigen-specific proliferative T cell responses by reducing the antigen presentation capacity of dendritic cells (DCs), monocytes, macrophages, and other antigen-presenting cells (APCs) through MHC class II downregulation,^6^ limiting co-stimulation, and production of inflammatory cytokines such as IL-12.^7^ Reduction in constitutive and IFNγ-induced MHC class II expression in monocytes leads to inhibition of antigen-specific T cell responses, partially contributing to tolerance of presented antigens, including tumor-derived antigens.

Among CD4^+^ T cells, the major producers of IL-10 are FoxP3^+^ regulatory T cell (Treg) cells and type 1 regulatory T (Tr1) cells.^8^^,^^9^ In Tr1 cells, IL-10 is co-expressed with lymphocyte-activation gene 3 (LAG3) and can be induced by the cytokine IL-27,^10^ which can in turn activate the transcription factor c-Maf to promote Tr1 cell differentiation.^11^ The Tr1 cell-associated markers T cell immunoreceptor with immunoglobulin and immunoreceptor tyrosine-based inhibitory motif [ITIM] domain (TIGIT) and LAG3 bind CD155 and MHC Class II respectively, interfering with optimal TCR transduction and immunological synapse formation.^12^^,^^13^ Egr-2^14^ in combination with Blimp-1^15^ is required to mediate IL-10 production in IL-27-activated murine CD4^+^ T cells.

IL-10 expression has also been linked to the relative strength of TCR signaling,^16^ and repeated dosing of Tg4 TCR transgenic mice with a modified self-peptide leads to the progressive emergence of FoxP3^−^ Tr1-like IL-10^+^ T cells.^17^ We recently developed an accelerated adaptive tolerance model^18^ that induces expression of *Il10*-enhanced green fluorescent protein (EGFP) within 24 h of primary *in vivo* immunization with a high dose of self-antigen.^19^ *Il10*-EGFP^+^ T cells arose from T cells experiencing the highest levels of TCR signal strength *in vivo* and expressed higher levels of LAG3 and TIGIT.^19^ Despite uniform activation of T cells in the accelerated adaptive tolerance model, as evidenced by activation of nuclear factor of activated T cells (NFAT)-driven *Nr4a3* transcription, only 15%–25% of activated T cells go on to transcribe *Il10*. This finding suggests that high antigen concentrations alone are not sufficient for promoting *Il10* transcription and that other factors and cellular interactions within the local milieu play important roles. Here, we established a model for the rapid induction of Tr1 cells *in vivo*. Our system revealed a non-redundant regulatory role for IFNγ in combination with IL-27 to promote Tr1 cell differentiation under conditions of strong tolerogenic TCR signaling.

## Results

### Kinetics of *de novo Il10* transcription in response to strong tolerogenic TCR signaling

**Figure 1:**
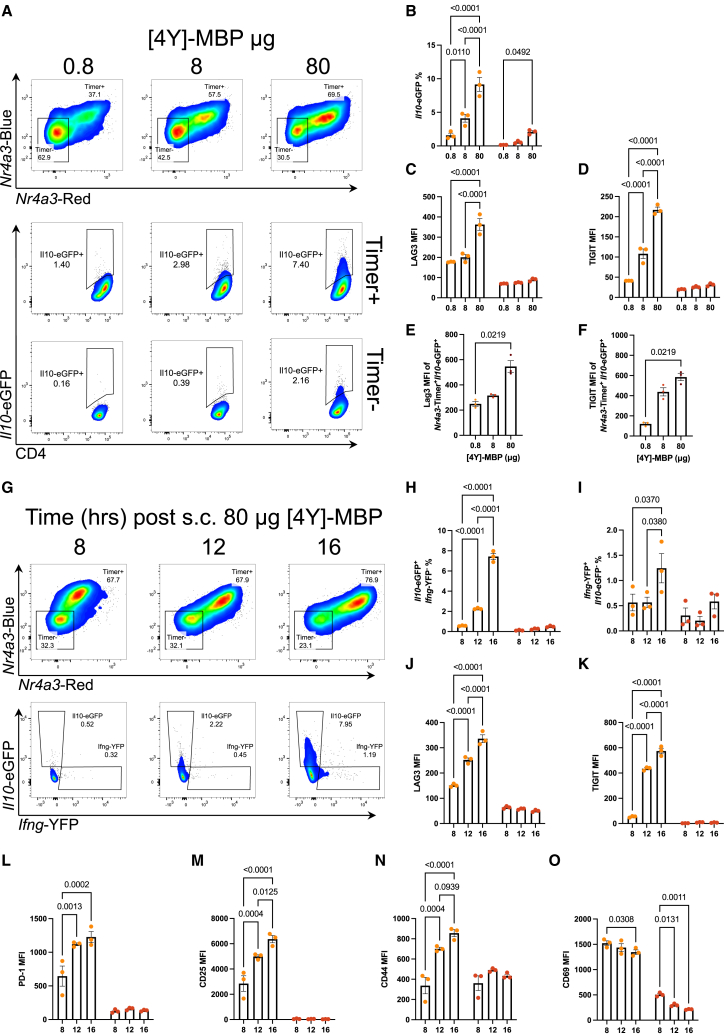
Kinetics of *de novo Il10* transcription in response to strong tolerogenic TCR signaling Tg4 *Nr4a3*-Tocky *Il10*-EGFP mice were immunized with 0.8, 8, or 80 μg [4Y]-MBP in 200 μL PBS subcutaneously (s.c.), and spleens were harvested at 24 h. Representative flow plots (A) showing CD4^+^ TCRvβ8.1/8.2^+^ according to *Nr4a3*-Timer expression above and the *Il10*-EGFP^+^ of *Nr4a3*-Timer^+^ and *Nr4a3*-Timer^−^ below. Summary of *Il10*-EGFP frequency (B), LAG3 MFI (C), and TIGIT MFI (D) in *Nr4a3*-Timer^+^ (orange) or *Nr4a3*-Timer^-^ (red). Summary of LAG3 (E) and TIGIT MFI (F) in *Il10*-EGFP^+^*Nr4a3*-Timer^+^. Tg4 *Nr4a3*-Tocky *Il10*-EGFP *Ifng*-YFP mice were administered 80 μg [4Y]-MBP in 200 μL PBS s.c., and spleens were harvested at 8, 12, or 16 h. Representative flow plots (G) showing CD4^+^ TCRvβ8.1/8.2^+^*Nr4a3*-Timer expression above and below *Il10*-EGFP against *Ifng*-YFP of *Nr4a3*-Timer^+^ below. Summary of *Il10*-EGFP^+^*Ifng*-YFP^−^ (H) and *Ifng*-YFP^+^*Il10*-EGFP^−^ (I) frequency, LAG3 (J), TIGIT (K), PD-1 (L), CD25 (M), CD44 (N), and CD69 (O) MFI in *Nr4a3*-Timer^±^. (B–F and H–O) bars represent mean ± SEM. Statistical analysis by two-way ANOVA with Tukey’s multiple comparisons test (B–D and H–O) or Kruskal-Wallis test with Dunn’s multiple comparisons. *n* = 3 per treatment or time.

We previously reported that strong TCR signaling drives *Il10* transcription.^19^ To further investigate the development of *Il10*^+^ T cell populations and their relationship to Tr1 cells in this model, we performed tolerogenic immunization of Tg4 *Nr4a3*-Tocky *Il10*-EGFP transgenic mice with a single dose of tyrosine at position 4 [4Y] myelin basic protein (MBP) peptide. Firstly, we established baseline expression of downstream TCR signaling (*Nr4a3*-Timer, hereafter referred to as “Timer”) and *Il10*-EGFP reporters under a low, medium, and high (0.8, 8, and 80 μg) dose of [4Y]-MBP peptide at 24 h. As *Nr4a3*-Timer expression is downstream of TCR engagement and NFAT signaling,^20^ we used it as a proxy marker for TCR-specific activation. Timer^+^ population positively correlated with [4Y]-MBP peptide dose (Figure 1A) and established that *Il10*-EGFP was almost exclusively found within the Timer^+^ fraction (i.e., recently TCR signaled, Figure 1B). In the Timer^+^ population, LAG3 (Figure 1C) and TIGIT (Figure 1D) positively correlated with peptide dose only within the Timer^+^ fraction, confirming the importance of TCR signaling strength for induction of IL-10, LAG3, and TIGIT in this model. Furthermore, we also observed dose-dependent increases in the levels of LAG3 (Figure 1E) and TIGIT (Figure 1F) within *Il10*-EGFP^+^ T cells. Furthermore, at the highest immunization dose, *Il10*-EGFP was co-expressed with the Tr1 markers Lag3 and Tigit (Figure S1), and most *Il10*-EGFP^+^ T cells did not express Foxp3 (Figure S2).

IL-10 expression has been previously observed in the Tg4 tolerance model to arise from IFNγ-producing Th1 cells that have undergone repeated stimulation.^8^ To assess the relationship between the development of *Il10*-EGFP and IFNγ production, we performed kinetic analysis at 8, 12, and 16 h following high dose of [4Y]-MBP (80 μg) stimulation of Tg4 *Nr4a3*-Tocky *Il10*-EGFP *Ifng*-yellow fluorescent protein (YFP) transgenic mice. Timer expression shifts from mostly “blue^+^” to mostly “blue^+^ red^+^” over the time course (Figure 1G, top panel), representing a shift from recently activated (<4 h) to persistent activation (>4 h, <24 h). *Il10*-EGFP and *Ifng*-YFP are also increasingly expressed within this Timer^+^ population and predominantly arise independently of each other (Figure 1G, bottom panel). *Il10*-EGFP significantly and steadily increases between 8 and 16 h in the Timer^+^ population (Figure 1H), with no significant increases in *Il10*-EGFP for the Timer^−^ fraction. Within CD4^+^ T cells, *Ifng*-YFP is expressed in both Timer^+^ and Timer^−^ at all time points, with a small rise at 16 h (Figure 1I). In the Timer^+^ cells, LAG3, TIGIT, PD-1, CD25, and CD44 (Figures 1J–1N, respectively) showed increasing expression from 8 to 16 h. Notably, CD69 did not follow this pattern, showing largely stable expression in both Timer positive and negative populations (Figure 1O), implying this marker fails to capture TCR signaling dynamics in this model. These findings demonstrate that CD4^+^ T cells can rapidly transcribe *Il10* and increase expression of Tr1 markers in a TCR signal strength- and time-dependent fashion during the first 24 h of immunization.

### Rapidly induced *Il10*-expressing T cells are Tr1-like cells and have a transcriptionally delayed program

**Figure 2:**
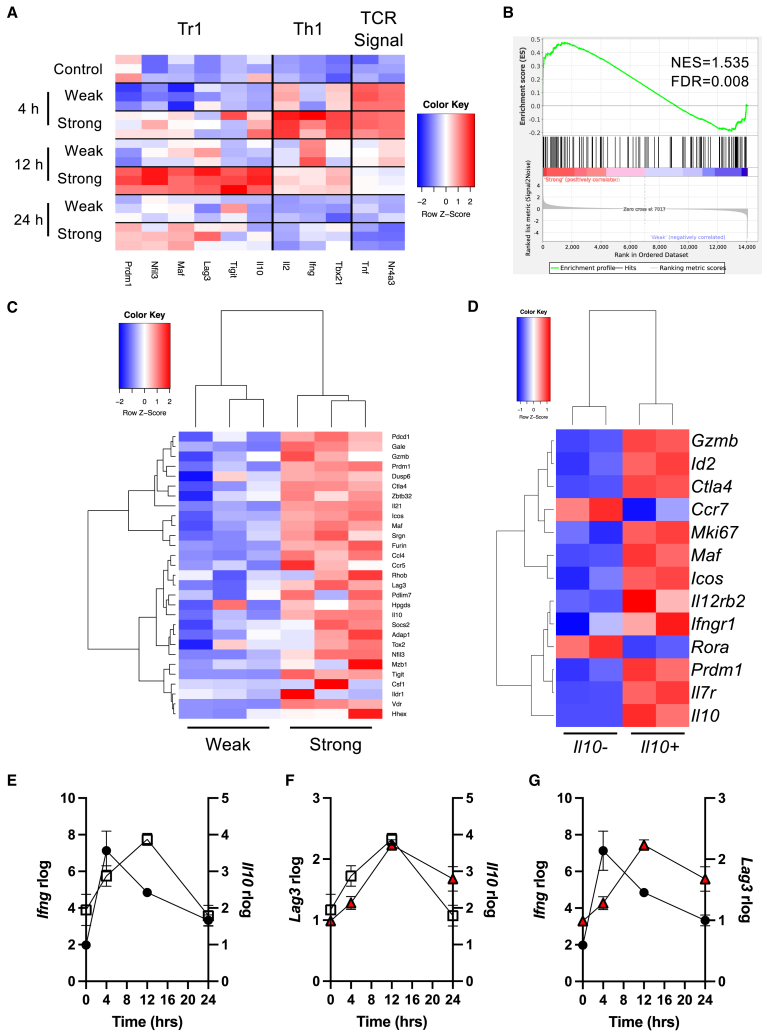
Rapidly induced *Il10*-expressing T cells are Tr1-like cells and have a transcriptionally delayed program (A) Re-analysis of GEO: GSE165817 (Elliot et al.^19^) where Tg4 *Nr4a3*-Tocky *Il10*-EGFP mice were administered with 0 (control), 8, or 80 μg [4Y]-MBP in 200 μL PBS s.c. and spleens harvested at 4, 12, and 24 h for bulk RNA isolation and transcriptome analysis. Heatmap of markers associated with early T cell activation, Th1 phenotype or Tr1-like cells arising after expression of Th1 cell hallmarks. (B) Gene set enrichment analysis (GSEA) using Tr1 genes defined in Chen et al.^21^ comparing 12 h gene expression in 80 μg vs. 0.8 μg immunized mice. (C) Heatmap of rlog-normalized data for GSEA core-enrichment genes for weak (0.8 μg) versus strong (80 μg) conditions. Tg4 *Nr4a3*-Tocky *Il10*-EGFP mice were administered 4 mg/kg [4Y]-MBP in 200 μL PBS s.c., and 24 h later CD4^+^*Il10-*EGFP+ and CD4^+^*Il10*-EGFP^−^ T cells were sorted for analysis by mRNA-seq. (D) Transcriptome analysis of CD4^+^*Nr4a3*-Timer^+^*Il10*-EGFP^±^ as a heatmap of curated Tr1 differentially expressed genes (DEGs) between conditions at 24 h. (E–G) Compared temporal expression rlog transcripts from GEO: GSE165817 (Elliot et al.^19^) for *Ifng* against *Il10* (E), *Ifng* against *Lag3* (F), and *Lag3* against *Il10* (G) from Tg4 *Nr4a3*-Timer *Il10*-EGFP mice administered with 80 μg [4Y]-MBP in 200 μL PBS s.c. and mRNA transcripts depicted at 4, 12, and 24 h.

To understand the kinetics of development of the Tr1 transcriptional program, we re-analyzed a past RNA sequencing (RNA-seq) time course experiment performed on CD4^+^ T cells that had received 0 (control), 0.8, or 80 μg [4Y]-MBP for up to 24 h *in vivo*.^19^ Here we initially focused on markers associated with TCR signaling, Th1 cells, and Tr1 phenotype (Figure 2A). Our analysis showed that strong tolerogenic stimulation led to the rapid activation of the early activation genes *Nr4a3* and *Tnf*, which was also linked to a rapid transcriptional burst of *Tbx21*, *Ifng*, and *Il2* (Th1-type signature) at 4 h. The Tr1 module (comprising *Il10*, *Tigit*, *Lag3*, *Maf3*, *Nfil3*, and *Prdm1*) appeared delayed and arising more uniformly at 12 h and was only visible in the condition of strong TCR signaling. We performed gene set enrichment analysis (GSEA) on the 12 h 80 μg vs. 0.8 μg for Tr1 cell phenotype using a previously published gene set for human Tr1 cell, revealing a strong enrichment for the Tr1 cell phenotype (normalized enrichment score [NES] = 1.535, false discovery rate = 0.008) (Figure 2B). Heatmap of the leading-edge genes in the GSEA clearly showed the effect of TCR signal strength on Tr1 gene signatures (Figure 2C).

To confirm that the *Il10*-EGFP^+^ T cells represented Tr1 cells, we sorted CD4^+^ *Il10*-EGFP^+^ and CD4^+^ *Il10*-EGFP^−^ T cells to undergo mRNA sequencing (mRNA-seq) analysis at 24 h (Figure 2D). Heatmap analysis revealed that the *Il10-*EGFP^+^ subset was enriched with transcripts for hallmark signatures of Tr1 cells, including *Maf*, *Prdm1* (encoding Blimp-1), *Il10*, and *Ctla4*. In addition, they also showed enhanced expression of *Il12rb2* and *Ifngr1*, both of which encode receptors important in the generation of Th1 responses.

Given the potential relevance of the early type 1 immune signature, we compared the transcriptional changes at 4, 12, and 24 h time points post-peptide administration for *Ifng*, *Il10*, and *Lag3* (Figures 2E–2G). Analysis confirmed that the *Ifng* transcript peak preceded the *Il10* transcriptional peak by around 8 h, with *Ifng* peaking at 4 h and *Il10* at 12 h (Figure 2E), although analysis in Figure 1 suggested that this expression was likely from distinct CD4^+^ T cell subsets. *Lag3* transcriptional peak also showed a similar relationship to *Ifng* as *Il10* (Figure 2F), which was clearly observed when *Il10* and *Lag3* transcriptional kinetics were compared head to head (Figure 2G). In addition, given the transient activation of the Tr1 gene family, we evaluated the persistence of the Tr1 cell state *in vivo* (Figure S3). Here we saw a sharp decline by 72 h in Tr1 cell frequency (Figure S3A) and expression of markers of TCR signaling (Figures S3B and S3C). Finding that a non-Tr1 CD4^+^ T cell *Ifng* transcriptional burst preceded the emergence of Tr1 cells in our accelerated adaptive tolerance model, we hypothesized that it may play a role in regulating Tr1 cell development.

### IFNγ positively regulates Tr1 cell differentiation

Having established that *Il10*-EGFP is expressed by rapidly induced Tr1 cells, and the *Il10* transcript is preceded by *Ifng* transcript, we investigated *in vitro* whether addition of IFNγ would enhance *Il10-*EGFP^+^ T cell frequency upon activation of CD4^+^ T cells *in vitro* (Figure S4). These data confirmed that addition of IFNγ had no direct effect on *Il10*-EGFP expression.

**Figure 3:**
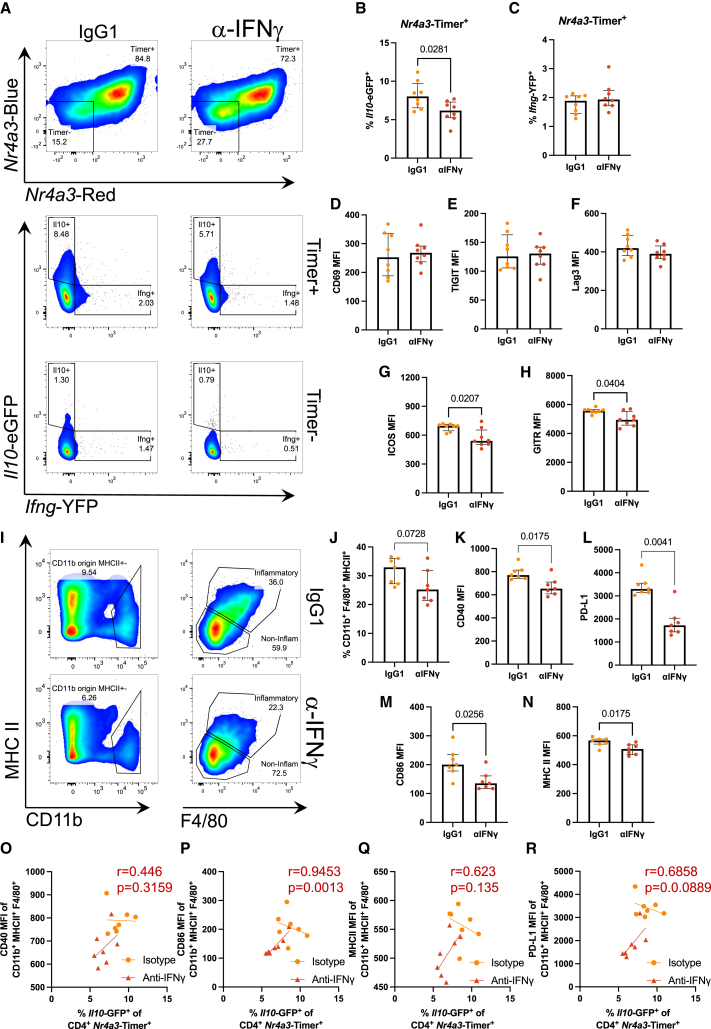
Interferon-gamma positively regulates Tr1 cell differentiation Tg4 *Nr4a3*-Tocky *Il10*-EGFP *Ifng*-YFP mice were immunized with 4 mg/kg [4Y]-MBP in PBS s.c. and 1 mg αIFNγ or IgG1 isotype in 200 μL PBS i.p., and spleens were harvested 24 h later. Representative flow plots at 24 h (A) showing CD4^+^ TCRvβ8.1/8.2^+^ according to *Nr4a3*-Timer expression above and the *Il10*-EGFP against *Ifng*-YFP of *Nr4a3*-Timer^+^ below. Summary of *Il10*-EGFP (B) and *Ifng*-YFP (C) frequency in *Nr4a3*-Timer^+^ and CD69 (D), TIGIT (E), LAG3 (F), ICOS (G), and GITR (H) MFI. Tg4 *Nr4a3*-Tocky *Il10*-EGFP *Ifng*-YFP mice were immunized with 4 mg/kg [4Y]-MBP in PBS s.c. and 1 mg αIFNγ or IgG1 isotype in 200 μL PBS i.p., and spleens were harvested at 12 h (I) showing CD11b against MHC Class II to the left, and from CD11b^+^, F4/80 against MHC Class II to the right. Summary of F4/80^+^ MHCII^+^ (“Inflammatory” myeloid) frequency (J) CD40 (K), PD-L1 (L), CD86 (M), and MHC Class II (N) MFI. Linear regression (Pearson’s correlation *p* value and r value in red) of CD4^+^*Nr4a3*-Timer^+^*Il10*-EGFP^+^ frequency against CD40 (O), CD86 (P), MHC Class II (Q), and PD-L1 (R) MFI in the inflammatory myeloid compartment. (B–H and I–N) Bars represent median with interquartile range. Statistical analysis by Mann-Whitney U test. *n* = 8 (A–I) and *n* = 7 (J–R) per treatment.

Next, we addressed the role of IFNγ *in vivo*: we administered αIFNγ antibody before weight-normalized immunization of Tg4 *Nr4a3*-Tocky *Il10*-EGFP *Ifng-*YFP *mice* with 4 mg/kg of [4Y]-MBP peptide. At 24 h, splenic CD4^+^ T cell responses were analyzed by flow cytometry (Figure 3A). Analysis of Tg4 *Nr4a3*-Timer^+^ T cells revealed that the frequency of *Il10*-EGFP (Figure 3B) but not *Ifng*-YFP (Figure 3C) expressers was significantly reduced by αIFNγ treatment. αIFNγ treatment did not alter CD69, TIGIT, or LAG3 expression (Figures 3D–3F) but did significantly reduce inducible T cell costimulator (ICOS) and glucocorticoid-induced TNFR family related gene (GITR) (Figures 3G and 3H), which we have previously shown to be markers of TCR signal strength in this model.^19^ It also had only a very minor effect on *Il10*-EGFP^+^ T cells within the Timer^−^ CD4^+^ T cell fraction (Figure S5).

Given the changes in *Il10* and markers of TCR signal strength, we hypothesized that IFNγ may have a role in augmenting TCR signal strength and hence the induction of Tr1 cells in our model. We performed analysis of splenic DC, B cell, and macrophage subsets. Analysis revealed a general reduction in CD19^−^ MHC class II^+^ cells within the splenic environment but had no impact on DC frequency but did reduce DC levels of PD-L1 (Figure S6). Analysis of splenic macrophages, identified as MHC class II^+^ F4/80^+^ cells from a CD11b^+^ origin^22^ (Figure 3I), revealed a strong trend for a reduction in this population (Figure 3J) and that these splenic macrophages all had significantly reduced levels of CD40 (Figure 3K), PD-L1 (Figure 3L), CD86 (Figure 3M), and MHC class II (Figure 3N). Correlation analysis of the frequency of *Il10-*EGFP^+^ T cells in comparison to macrophage activation status highlighted that, within αIFNγ treated groups, all showed positive correlations (Figures 3O–3R) with CD86 significantly and strongly correlation with Tr1 cell frequency. These data demonstrate that IFNγ plays a positive regulatory role in enhancing Tr1 cell development, which is correlated with reduced levels of TCR signal strength and myeloid cell activation.

### Anti-IFNγ treatment reduces TCR signal strength *in vivo*

**Figure 4:**
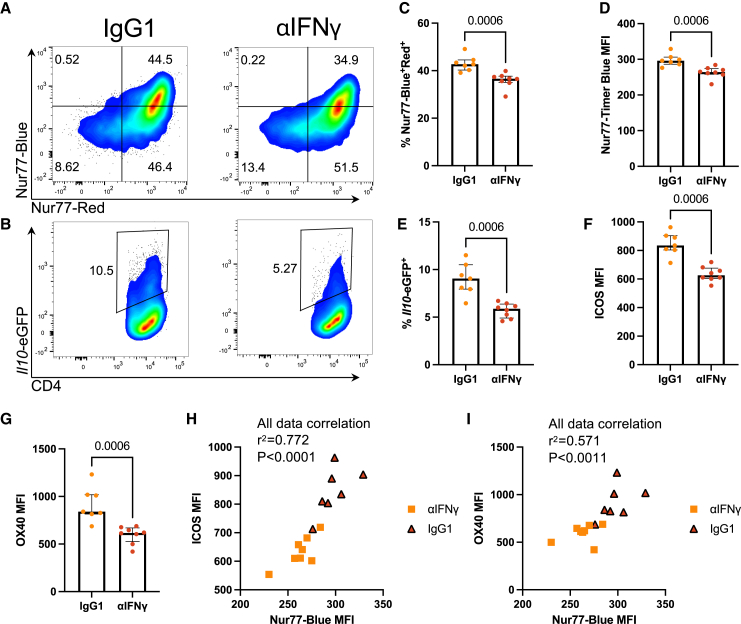
Anti-IFNγ treatment reduces TCR signal strength *in vivo* Tg4 Nur77-Tempo *Il10*-EGFP mice received 1 mg αIFNγ or IgG1 isotype in 200 μL PBS i.p before immunization with 4 mg/kg [4Y]-MBP in PBS s.c., and spleens were harvested at 24 h. (A and B) (A) Nur77-Blue versus Nur77-Red or (B) *Il10*-EGFP vs. CD4 in CD4^+^ T cells at 24 h. (C–I) (C) Summary of % Nur77-Blue^+^Red^+^, (D) Nur77-Blue MFI in the two treatment, or % *Il10*-EGFP (E) in CD4^+^ T cells. ICOS (F) or OX40 (G) MFI in CD4^+^ T cells. Correlation of Nur77-Blue MFI versus ICOS MFI (H) or OX40 MFI (I) in CD4^+^ T cells using linear regression (Pearson’s correlation p value and r^2^ values are shown). Isotype (*n* = 7), αIFNγ (*n* = 8). Bars represent median+/−interquartile range (IQR). Statistical analysis by Mann-Whitney U test.

To determine that IFNγ was modulating the perceived TCR signal strength *in vivo*, we used the TCR signal strength-sensitive marker Nur77.^23^ We employed our Nur77-Tempo mice, which are more sensitive than *Nr4a3*-Tocky to subtle changes in TCR signal strength.^24^ We administered αIFNγ antibody before immunizing Tg4 Nur77-Tempo *Il10*-EGFP *Ifng-*YFP mice with 4 mg/kg of [4Y]-MBP peptide. We then analyzed the expression of Nur77-Blue versus Nur77-Red (Figure 4A) and Tr1 cell frequency (Figure 4B). IFNγ neutralization led to a reduction in Nur77-Blue^+^Red^+^ T cells (Figure 4C) and a significant reduction in Nur77-Timer Blue MFI *in vivo* (Figure 4D), indicating that IFNγ modulates TCR signal strength *in vivo*. In addition, as before, IFNγ neutralization led to a significant reduction in Tr1 cells (Figure 4E). In addition, we saw a significant reduction in ICOS and OX40 (Figures 4F and 4G), whose expression patterns across both treatment groups collectively positively correlated with Nur77-Blue levels, highlighting that ICOS and OX40 expression levels are proxies for TCR signal strength in this model (Figures 4H and 4I). In summary, reduction in Tr1 cells by anti-IFNγ treatment is correlated with a reduction in TCR signal strength.

### Natural killer and T cells are major sources of IFNγ, but natural killer cells are redundant for Tr1 cell induction

**Figure 5:**
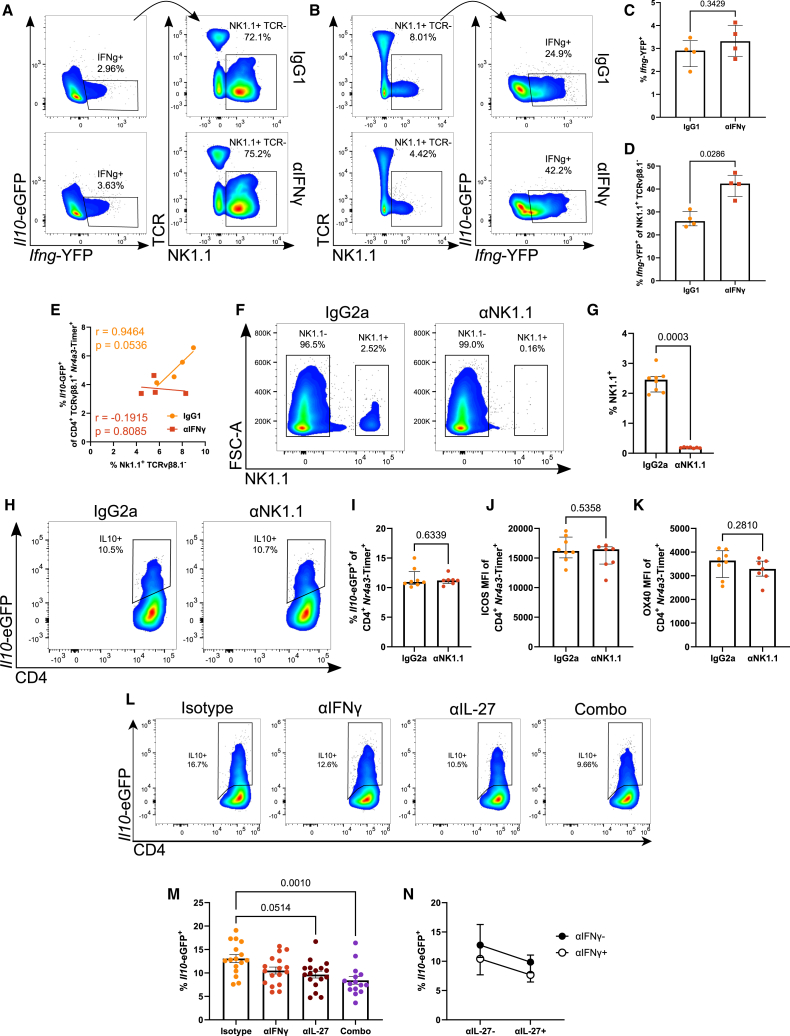
IFNγ and IL-27 additively regulate Tr1 cell development Tg4 *Nr4a3*-Tocky *Il10*-EGFP *Ifng*-YFP mice were administered 4 mg/kg [4Y]-MBP in PBS s.c. and 1 mg αIFNγ or IgG1 isotype in 200 μL PBS i.p., and spleens were harvested at 24 h. Representative flow plots showing *Il10*-EGFP against *Ifng*-YFP above and *Ifng*-YFP^+^-derived TCRvβ8.1/8.2 against NK1.1 below (A) and TCRvβ8.1/8.2 against NK1.1 above and NK1.1^+^-derived *Il10*-EGFP against *Ifng*-YFP below (B). Summary of *Ifng*-YFP^+^ frequency (C) from (A) and *Ifng*-YFP^+^ frequency from NK1.1^+^ (D) from (B). Linear regression (Pearson’s correlation) of CD4^+^*Nr4a3*-Timer^+^*Il10*-EGFP^+^ against NK1.1^+^ TCRvβ8.1/8.2^−^ (E). Tg4 *Nr4a3*-Tocky *Il10*-EGFP mice were given 200 μg αNK1.1 or IgG2a isotype in 200 μL PBS i.p. and 48 h later were immunized with 4 mg/kg [4Y]-MBP in PBS s.c., and spleens were harvested 24 h later. Representative spectral cytometry plots showing NK1.1 against FSC-A (F). Summary of NK1.1^+^ (G) from (F). Representative spectral cytometry plots at 24 h showing *Il10*-EGFP against CD4 (H). Summary of *Il10*-EGFP^+^ frequency (I) and ICOS (J) and OX40 (K) MFI of CD4^+^*Nr4a3*-Timer^+^ T cells. Tg4 *Nr4a3*-Tocky *Il10*-EGFP mice were administered 4 mg/kg [4Y]-MBP in PBS s.c. and 0.5 mg αIFNγ, αIL-27, a combination of both, or a mixed IgG1 and IgG2a isotype in 200 μL PBS i.p., and spleens were harvested 24 h later. Representative spectral cytometry plots at 24 h showing *Il10*-EGFP against CD4 in *Nr4a3*-Timer^+^ (L). Summary of *Il10*-EGFP^+^ frequency within the CD4^+^*Nr4a3*-Timer^+^ T cells. (M) Analysis for additive effect of treatment by factoring αIFNγ and αIL-27 as two variables (N).(C, D, G, I–K, M, and N) Bars represent median with interquartile range. Statistical analysis by Mann-Whitney U test (C, D, and I–K), Kruskal-Wallis test with Dunn’s multiple comparisons (M). *n* = 4 (A–E), *n* = 8 (F–K), *n* = 15 (Combo), *n* = 16 (isotype), *n* = 17 (αIFNγ), and *n* = 17 (αIL-27) (L–N) per treatment.

Next, we wanted to determine the major splenic sources of IFNγ *in vivo*. Following immunization of Tg4 *Nr4a3*-Tocky *Il10*-EGFP *Ifng-*YFP mice and treatment with isotype or αIFNγ, we further analyzed the *Ifng*-YFP^+^ compartments (Figure 5A). NK1.1^+^ TCR^−^ cells (Figure 5A) comprised most of the *Ifng-*YFP producers, with the remainder being largely T cell derived. Interestingly, total splenic frequency of *Ifng*-YFP^+^ cells was unaffected by αIFNγ treatment but did drive a significant increase in *Ifng*-YFP levels in NK1.1^+^ cells (Figures 5B–5D).

We were interested to understand relationships between natural killer (NK) cells and the development of Tr1 cells (identified as *Il10-*EGFP^+^ T cells). CD4^+^ Timer^+^ *Il10*-EGFP^+^ T cells directly correlate with the NK1.1^+^ TCR^−^ population under isotype treatment, but αIFNγ decouples this, removing the correlation between them (Figure 5E). Based on this, we hypothesized that NK1.1^+^ cells, as major *Ifng*-YFP expressors, may be the important physiological source of IFNγ that contributes to the regulation of Tr1 cell development. To determine the functional role of NK cells, we performed antibody depletion of NK1.1 cells before immunization with [4Y]-MBP to induce Tr1 cells *in vivo*. Spectral cytometry confirmed staining for NK1.1^+^ (using a different antibody clone) that very few NK1.1^+^ cells remained following administration of the depleting antibody (Figures 5F and 5G). However, NK cell depletion had no effect on the frequency of *Il10-*EGFP^+^ T cells (Figures 5H and 5I) nor on markers of strong TCR signaling ICOS (Figure 5J) or OX40 (Figure 5K). These data therefore suggest that NK cell IFNγ is redundant for Tr1 cell development in this model and suggests that T cell- or NKT cell-derived IFNγ may be the more important source.

### IFNγ and IL-27 additively regulate Tr1 cell development

IL-27 is a potent, innate immune cell-derived inducer of IL-10 production.^11^ We were interested to compare the relative potency of IL-27 versus IFNγ for the induction of Tr1 cells in our accelerated adaptive tolerance model. We administered αIFNγ and/or αIL-27 along with immunization of Tg4 *Nr4a3*-Tocky *Il10*-EGFP mice with 4 mg/kg [4Y]-MBP to examine the effect on Tr1 cell development (Figures 5L and 5M). In this setting, co-blockade of IL-27 and IFNγ resulted in a highly significant reduction in Tr1 cell frequency (Figure 5M), and analysis showed that this effect appeared to be additive when visualizing αIL-27 and αIFNγ treatment as factored variables (Figure 5N).

## Discussion

Negative feedback control of T cell responses by IL-10 is important in regulating Th1 cells that have undergone repeated stimulation.^8^ In our study we have demonstrated that the Th1-associated cytokine IFNγ indirectly promotes *Il10* expression in Tr1 cells through enhancing TCR signaling, highlighting a feedback loop that may act to control immunopathology. IL-10 increased IFNγ and FasL in TCR-stimulated CD8^+^ T cells *in vitro.*^25^ However, it also activates NK cells with increased cytotoxic function.^26^^,^^27^ Despite this, IL-10 has been known to inhibit IFNγ production by NK cells in the presence of APCs, partially due to a decrease in IFNγ-inducing cytokines.^3^^,^^28^ This observation is consistent with our finding that, under conditions of αIFNγ treatment, which lowers Tr1 cell frequency, we saw a concomitant increase in NK cell IFNγ. While in this model we ruled out NK cell IFNγ as the source, it does not exclude a potential role for other immune cell subsets such as NK T cells or innate lymphoid cells (ILCs). In addition, it is possible that NK cells are positive for transcript for *Ifng* (and hence would appear YFP^+^) but do not produce protein.

Thymic FoxP3^+^ Tregs comprise a self-reactive polyclonal T cell population that undergoes thymic selection in response to strong and persisting TCR signaling.^23^^,^^29^ Likewise, Tr1 cells have been previously reported to progressively develop by strong^19^ and/or chronic^16^^,^^17^ antigenic stimulation in the periphery.^9^ *Il10*^+^ T cells induced by strong TCR signaling in our accelerated adaptive tolerance model are highly refractory to re-stimulation^19^; we have previously suggested that IL-10 marks T cells in an acute state of negative feedback.^19^ Mechanistically this has been linked to chromatin remodeling of regulatory gene regions in tolerogenic T cells, whereby anti-inflammatory regions remain accessible to sub-threshold TCR signals while inflammatory gene loci are silenced.^30^ Induced Tr1 cells and peripheral FoxP3^+^ Treg may share some evolutionary redundant functions, since genetic deletion of the conserved non-coding DNA sequence one (CNS1) of the *Foxp3* gene leads to loss of FoxP3^+^ T cells within the mesenteric lymph node but is compensated for by an expansion of Tr1-like IL-10^+^ T cells.^31^ This suggests that IL-10^+^ T cell subsets may be able to compensate for each other in some circumstances, e.g., at environmental interfaces where IL-10 is critical.^32^

IL-10 is elicited preferentially in Tr1 cells when they are strongly stimulated through their TCR under tolerogenic conditions.^30^ Clearly, however, TCR signaling alone is unlikely to be the sole promoting factor for IL-10 expression, and other signals are likely to contribute. Tolerogenic stimulation, however, is likely to provide a different activation pattern of nuclear transcription factors than T cells activated in the context of enhanced co-stimulatory molecule expression. Many features of so called “exhausted” T cells or Tr1 cells have been linked to chromatin remodeling.^30^^,^^33^ The relative activity of NFAT alone versus NFAT: activator protein-1 (AP-1) complexes is thought to be important in co-ordinating the dysfunctional T cell program,^30^^,^^33^ and it will be interesting to explore whether similar mechanisms are involved in the differentiation of Tr1-like cells under tolerizing conditions. It is interesting to note that IFNγ neutralization reduced expression of the TCR-signal-strength-dependent marker Nur77, showing that, even under tolerogenic stimulation conditions, IFNγ can influence the magnitude of TCR signaling. This effect may be in part due to upregulation of co-stimulatory molecules such as CD86, which may further supplement strong TCR signals.

It is intriguing that *Il10* can be rapidly (within 4 to 12 h) transcribed in response to a single dose of soluble peptide and suggests that the *Il10* locus can be rapidly remodeled within the CD4^+^ T cell population. As to whether this is unique to the subset transcribing *Il10* or is a general feature of a population of T cells that have been strongly stimulated through their TCR remains to be determined. It also raises the question as to whether further heterogeneity exists within the CD4^+^ T cell population that may pre-dispose some populations to *Il10* transcription over others (e.g., potential memory-like subsets or T cells experiencing different grades of tonic signal in different environments^24^). It is also clear from our data that aspects of the Tr1 transcriptional signature generated through our model are dynamic with some signature genes transcriptionally dampened 24 h after the primary immunization.^19^ By 72 h there was a notable decline in Tr1 cell markers. This may reflect that, at least in these models, the Tr1 phenotypes reflect CD4^+^ T cells that have undergone recent and relatively strong TCR signaling in the absence of major co-stimulation.^19^

Our findings that macrophage activation status directly correlated with the emergence of IL10^+^ T cell expression suggests that, aside from their increased antigen-presenting capacity due to IFNγ priming, they may also provide additional signals. For this reason, we explored the potential role of IL-27 in our model. IFNγ and MYD88 are known to upregulate IL-27 within macrophages,^34^ so it is possible that IFNγ may work through both augmentation of TCR signal strength and the induction of the Tr1-promoting cytokine IL-27. It remains to be determined how the inclusion of innate pathogen recognition receptor signals would alter the differentiation of T cells experiencing strong TCR signaling within the immune synapse. In addition, activation of APCs would lead to production of T helper cell polarizing cytokines that may influence the activation of transcription factors to promote alternative T helper cell differentiation pathways, such as the Th1 pathway. We observed a small additive effect when we neutralized both IL-27 and IFNγ (almost halving the development of Tr1 cells *in vivo*), which suggests that their mechanisms of action may not be entirely overlapping.

It is well established that IL-10 expression is a signature cytokine of the effector Treg program,^35^ which is initiated by TCR signaling in Foxp3^+^ Treg cells.^36^ This strongly suggests that the *Il10* region is rapidly responsive to TCR stimulation in differentiated Treg, and *in vivo* αCD3 stimulation can enhance IL-10 expression *in vivo* during steady state.^37^ In a *T. gondii* infection model, IFNγ and IL-27 have been shown to promote CXCR3 and T-bet in Tregs but by separate mechanisms at separate sites—the periphery and musca, respectively.^38^ They also found that Tregs exposed to either IFNγ or IL-27 have distinct transcriptional profiles. Whether the local concentrations of these cytokines may also skew the phenotype of Tr1 cells to promote their recruitment to different anatomical sites remains to be explored.

In summary, our study has established that Tr1 cells can be rapidly generated *in vivo* in response to strong tolerogenic signaling. Our data support that local IFNγ and IL-27 combine to additively regulate their generation *in vivo*, highlighting a new “regulatory” role for IFNγ in the induction of tolerogenic Tr1 cells.

### Limitations of the study

While we have shown that IFNγ positively regulates Tr1 cell development and influences TCR signal strength, the precise cellular mechanism/s and important sources of IFNγ remain to be fully determined. Use of genetic tools to selectively delete IFNγ receptor on T cells would be required to fully evaluate a potential direct effect. Secondly, while NK cell depletion did not alter Tr1 cell frequency, other potential sources of IFNγ, such as ILCs and NK T cells, have not been evaluated in this study. Finally, while transcriptional analysis shows that the “Th1” transcriptional program is triggered early in CD4^+^ T cells, cytokine reporter data suggest that this likely arises from non-Tr1 cell populations and the kinetics of protein expression may differ.

## Resource availability

### Lead contact

Request for further information, resources, and reagents can be directed to the lead contact, Dr. David Bending, d.a.bending@bham.ac.uk.

### Materials availability

This study did not generate novel reagents.

### Data and code availability

Data for RNA-seq are deposited at GEO: GSE283286. Data from Elliot et al. 2021^19^ are available at GEO: GSE165817.

## Acknowledgments

This work was funded by the 10.13039/100010269Wellcome Trust MIDAS 4-year PhD program (D.A.J.L.), 10.13039/100010269Wellcome Trust Seed Award in Science (214018/Z/18/Z, D.B.), the Lister Institute of Preventive Medicine (D.B., S.T.R., and A.C.), and MRC Career development awards (MR/V009052/1, D.B. and L.S.; MR/S024611, R.A.D.). L.S.G. and D.C.W. are funded by the 10.13039/501100000855University of Birmingham. We thank the Tech Hub flow cytometry team led by Dr Guillaume Desanti and Ferdus Sheik at the University of Birmingham.

## Author contributions

D.B., L.S., and D.A.J.L. conceived and designed experiments and performed analysis. L.S., D.A.J.L., S.T.R., and L.S.G. performed experiments. D.B. performed RNA-seq analysis and GSEA analysis. R.A.D. provided expert advice on macrophage and DC phenotyping. D.C.W. provided expertise on models of adaptive tolerance. D.A.J.L. and D.B. wrote the initial manuscript. D.A.J.L., A.C., and D.B. reviewed and edited the manuscript. D.B. contributed to project funding acquisition, supervision, and management.

## Declaration of interests

The authors declare no competing interests.

## STAR★Methods

### Key resources table

**Table:** 

REAGENT or RESOURCE	SOURCE	IDENTIFIER
**Antibodies**		

Rat Anti-Mouse CD19 PerCP-Cy5.5 (clone 6D5)	BioLegend	Cat #115533; RRID:AB_2259869
Rat Anti-Mouse CD25 PerCP-Cy5.5 (clone PC61)	BioLegend	Cat #102030; RRID:AB_893288
Rat Anti-Mouse CD40 PerCP-Cy5.5 (clone 3/23)	BioLegend	Cat #124624; RRID:AB_2561474
Rat Anti-Mouse OX40 PerCP-Cy5.5 (clone OX-86)	BioLegend	Cat #119414; RRID:AB_2561724
Rat Anti-Mouse PD-1 PerCP-Cy5.5 (clone 29F.1A12)	BioLegend	Cat #135207; RRID:AB_10550092
Rat Anti-Mouse CD11b APC (clone M1/70)	BioLegend	Cat #101211; RRID:AB_312794
Armenian Hamster Anti-Mouse CD11c APC (clone N418)	BioLegend	Cat #117309; RRID:AB_313778
Rat Anti-Mouse CD25 APC (clone PC61)	BioLegend	Cat #102012; RRID:AB_312861
Armenian Hamster Anti-Mouse CD69 APC (clone H1.2F3)	BioLegend	Cat #104514; RRID:AB_492843
Rat Anti-Mouse FoxP3 APC (clone FJK-16s)	Thermo Fisher	Cat #17-5773-82; RRID:AB_469457
Rat Anti-Mouse LAG3 APC (clone C9B7W)	BioLegend	Cat #125209; RRID:AB_10639935
Rat Anti-Mouse OX40 APC (clone OX-86)	BioLegend	Cat #119413; RRID:AB_2561723
Rat Anti-Mouse PD-1 APC (clone 29F.1A12)	BioLegend	Cat #135210; RRID:AB_2159183
Rat Anti-Mouse PDL1 APC (clone 10F.9G2)	BioLegend	Cat #124311; RRID:AB_10612935
Rat Anti-Mouse/Human CD11b PE-Cy7 (Clone M1/70)	BioLegend	Cat #101215; RRID:AB_312798
Armenian Hamster Anti-Mouse CD11c PE-Cy7 (Clone N418)	Biolegend	Cat #117317; RRID:AB_493569
Rat Anti-Mouse CD19 PE-Cy7 (clone 6D5)	BioLegend	Cat #115519; RRID:AB_313654
Rat Anti-Mouse CD25 PE-Cy7 (clone PC61)	BioLegend	Cat #102015; RRID:AB_312864
Rat Anti-Mouse CD86 PE-Cy7 (clone GL-1)	BioLegend	Cat #105013; RRID:AB_439782
Rat Anti-Mouse ICOS PE-Cy7 (clone 7E.17G9)	BioLegend	Cat #117421; RRID:AB_2860636
Rat Anti-Mouse LAG3 PE-Cy7 (clone C9B7W)	BioLegend	Cat #125225; RRID:AB_2715763
Rat Anti-Mouse MHCII I-A/I-E PE-Cy7 (clone M5/114.15.2)	BioLegend	Cat #107629; RRID:AB_2290801
Mouse Anti-Mouse NK1.1 PE-Cy7 (clone S17016D)	BioLegend	Cat #156514; RRID:AB_2888852
Rat Anti-Mouse OX40 PE-Cy7 (clone OX-86)	BioLegend	Cat #119415; RRID:AB_2566154
Rat Anti-Mouse PD-1 PE-Cy7 (clone 29F.1A12)	BioLegend	Cat #135215; RRID:AB_10696422
Rat Anti-Mouse PDL1 PE-Cy7 (clone 10F.9G2)	BioLegend	Cat #124314; RRID:AB_10643573
Mouse Anti-Mouse TIGIT PE-Cy7 (clone 1G9)	BioLegend	Cat #142107; RRID:AB_2565648
Armenian Hamster Anti-Mouse CD11c AF700 (clone N418)	BioLegend	Cat #117319; RRID:AB_528735
Rat Anti-Mouse CD4 AF700 (clone RM4-4)	BioLegend	Cat #116022; RRID:AB_2715958
Armenian Hamster Anti-Mouse CD69 AF700 (clone H1.2F3)	BioLegend	Cat #104539; RRID:AB_2566304
Armenian Hamster Anti-Mouse ICOS AF700 (clone C398.4A)	BioLegend	Cat #313528; RRID:AB_2566126
Rat Anti-Mouse MHCII I-A/I-E AF700 (clone M5/114.15.2)	BioLegend	Cat #107621; RRID:AB_493726
Armenian Hamster Anti-Mouse TCRbeta AF700 (clone H57-597)	BioLegend	Cat #109224; RRID:AB_1027648
Armenian Hamster Anti-Mouse CD11c BUV395 (clone HL3)	BD Biosciences	Cat #564060; RRID:AB_2738580
Rat Anti-Mouse CD4 BUV395 (clone GK1.5)	BD Biosciences	Cat #563790; RRID:AB_2738426
Rat Anti-Mouse F4/80 BUV395 (clone T45-2342)	BD Biosciences	Cat #565614; RRID:AB_2739304
Mouse Anti-Mouse TCRVbeta8.1/8.2 BUV395 (clone MR5-2)	BD Biosciences	Cat #744335; RRID:AB_2742163
Rat Anti-Mouse CD11b BUV737 (clone M1/70)	BD Biosciences	Cat #612800; RRID:AB_2870127
Rat Anti-Mouse CD19 BUV737 (clone 1D3)	BD Biosciences	Cat #612782; RRID:AB_2870111
Rat Anti-Mouse CD4 BUV737 (clone GK1.5)	BD Biosciences	Cat #612761; RRID:AB_2870092
Rat IgG1 isotype (clone MAC 221)	Prof. Anne Cooke (University of Cambridge)	Gift from Prof. Anne Cooke (University of Cambridge)
Rat Anti-Mouse IFNγ (clone XMG1.2)	Prof. Anne Cooke (University of Cambridge)	Gift from Prof. Anne Cooke (University of Cambridge)
Mouse Anti-Mouse IL-27 p28 (clone MM27.7B1)	BioXCell	Cat #BE0326; RRID:AB_2819053
Mouse Anti-Mouse IgG2a (clone C1.18.4)	BioXCell	Cat #BE0085; RRID:AB_1107771
Anti-Mouse NK1.1 (clone PK136)	BioXCell	Cat #BE0036; RRID:AB_1107737

**Chemicals, peptides and recombinant proteins**		

MBP Ac1-9[4Y] peptide AcASQYRPSQR	GL Biochem Shanghai	Custom product
Phosphate Buffered Saline (Ca^2+^ Mg^2+^ free)	Thermo Fisher	Cat# 14190-094
RPMI 1640 with L-Glutamine	Thermo Fisher	Cat# 21875-034
DNAse I, Grade II	Roche	Cat# 10104159001
Collagenase D	Roche	Cat# 11088858001
Fetal Bovine Serum, qualified, heat inactivated, Brazil	Thermo Fisher	Cat# 10500064
Recombinant IFNγ	BioLegend	Cat# 714006

**Critical commercial assays**		

eFluor780 fixable viability dye	eBioscience	Cat# 65-0865-14
eBioscience™ Foxp3/Transcription Factor Staining Buffer Set	Thermo Fisher	Cat# 00-5523-00
eBioscience™ 1X RBC Lysis Buffer	Thermo Fisher	Cat# 00-4333-57

**Deposited Data**		

Sequencing data for *Il10*-eGFP^+^ versus *Il10*-eGFP^-^ CD4^+^ T cells	This study	GEO: GSE283286
Sequencing data for TCR signal strength analysis of murine T cells	Elliot et al.,^19^	GEO: GSE165817

**Experimental models: Organisms/Strains**		

Mouse: *Nr4a3*-Tocky Tg4 Tiger (*Il10*-GFP)	Elliot et al.^19^	Elliot et al.^19^
Mouse: Great (*Ifng*-YFP) Smart-17A	Price et al.^39^	Price et al.^39^
Mouse: *Il10*-GFP (Tiger)	Kamanaka et al.^37^	Kamanaka et al.^37^
Mouse: Nur77-Tempo	Elliot et al.^24^	EM:15078

**Software and algorithms**		

GraphPad Prism 10	GraphPad Inc	https://www.graphpad.com/scientificsoftware/prism/
FlowJo v10	BD Biosciences	https://www.flowjo.com/solutions/flowjo
Sony ID7000 Software	Sony Biotechnology	https://www.sonybiotechnology.com/us/instruments/id7000-spectral-cell-analyzer/software/
R version 4.0	R Core Team	https://www.r-project.org/
DESeq2	Love et al.^40^	Love et al.^40^
GSEA 4.3.3	UC San Diego and Broad Institute	https://www.gsea-msigdb.org/gsea/index.jsp

**Other**		

BD LSR Fortessa	BD Biosciences	Custom Product
BD FACS ARIA FUSION	BD Biosciences	Custom Product
ID7000 Spectral Cell Analyzer	Sony Biotechnology	Custom Product

### Experimental model and study participant details

#### Mice

Tg4 *Nr4a3*-Tocky *Il10*-eGFP mice were used as previously described.^19^ *Nr4a3*-Tocky Tg4 Tiger (*Il10*-eGFP) were mated to Great (*Ifng*-YFP) Smart-17A^39^ mice to generate *Nr4a3*-Tocky Tg4 Tiger (*Il10*-eGFP) Great (*Ifng*-YFP) Smart-17A mice. Nur77-Tempo mice^24^ were mated to Tg4 *Il10*-eGFP mice to generate Tg4 Nur77-Tempo *Il10*-eGFP lines. *Nr4a3*-Tocky mice^29^ were originally obtained under MTA from Dr Masahiro Ono, Imperial College London, UK. This manuscript does not report any findings arising from the use of unmodified *Nr4a3*-Tocky lines. All animal experiments were approved by the local animal welfare and ethical review body and authorised under the authority of Home Office licenses P18A892E0A and PP3965017 (held by D.B.). Animals were housed in specific pathogen-free conditions. Both male and female mice were used, and littermates of the same sex were randomly assigned to experimental groups.

### Method details

#### Immunisations

Tg4 *Nr4a3*-Tocky Tiger (*Il10*-eGFP) or Tg4 Nur77-Tempo *Il10*-eGFP mice were immunized through subcutaneous injection of [4Y]-MBP peptide (MBP Ac1-9[4Y] peptide AcASQYRPSQR, GL Biochem, Shanghai) with doses stated in figure legends in a total volume of 200 μL PBS. Mice were then euthanised at the indicated time points, and spleens removed to analyze systemic T-cell responses.

#### Antibody treatments

For *in vivo* blockade experiments, *in vivo* grade anti-IL-27p28 (clone MM27.7B1, mouse IgG2a, BioXcell), or anti-IFNγ (clone XMG1.2, kind gift from Prof Anne Cooke, University of Cambridge, rat IgG1) were administered through intraperitoneal injection as indicated in figure legends. For anti-IFNγ experiments, an isotype control group was used consisting of rat IgG1 (clone MAC221, kind gift from Prof Anne Cooke, University of Cambridge) and for anti-IL-27 experiments, an isotype control group was used consisting of mouse IgG2a (clone C1.18.4, BioXCell). For NK cell depletion, 200 μg of depleting NK1.1 (clone PK136, BioXCell) or mouse IgG2a (clone C1.18.4, BioXCell) was administered 48 hours before peptide immunisation.

#### CD4^+^ T cell isolation and culture

Naïve CD4^+^ T cells were isolated using MoJo magnetic bead negative selection kits (BioLegend) according to the manufacturer’s instructions. Naïve T cells were then cultured on 96 well flat bottom plates (Corning), which were pre-coated with 1 μg/mL anti-CD3 and 5 μg/mL anti-CD28, in 10% FBS (v/v) RPMI containing 1% penicillin/streptomycin (Life Technologies) at 37°C and 5% CO2 for the indicated time points in the presence of recombinant murine IFNγ (BioLegend)

#### Flow cytometry and cell sorting

For analysis of splenic lymphocytes single cell suspensions were prepared as previously described.^41^ For analysis of splenic myeloid populations spleens were dissociated using scissors in 1.2 mL of digestion media containing 1 mg/mL collagenase D (Merck Life Sciences) and 0.1 mg/mL DNase I (Merck Life Sciences) in 1 % FBS (v/v) RPMI. Samples were then incubated for 20-25 min at 37°C in a thermo-shaker. Digestion mixture was then passed through a 70 μm filter (BD Biosciences) and washed with 30 mL ice cold media (10 % FBS RPMI). Digested cells were washed once and stained in 96-well U-bottom plates (Corning). Analysis was performed on a BD LSR Fortessa X-20 instrument. The blue form of the Timer protein was detected in the blue (450/40 nm) channel excited off the 405 nm laser. The red form of the Timer protein was detected in the mCherry (610/20 nm) channel excited off the 561 nm laser. A fixable eFluor780-flurescent viability dye (eBioscience) was used for all experiments. Directly conjugated antibodies used in these experiments were: From BioLegend: Rat Anti-Mouse CD19 PerCP-Cy5.5 (clone 6D5), Rat Anti-Mouse CD25 PerCP-Cy5.5 (clone PC61), Rat Anti-Mouse CD40 PerCP-Cy5.5 (clone 3/23), Rat Anti-Mouse OX40 PerCP-Cy5.5 (clone OX-86), Rat Anti-Mouse PD-1 PerCP-Cy5.5 (clone 29F.1A12), Rat Anti-Mouse CD11b APC (clone M1/70), Armenian Hamster Anti-Mouse CD11c APC (clone N418), Rat Anti-Mouse CD25 APC (clone PC61), Armenian Hamster Anti-Mouse CD69 APC (clone H1.2F3), Rat Anti-Mouse LAG3 APC (clone C9B7W), Rat Anti-Mouse OX40 APC (clone OX-86), Rat Anti-Mouse PD-1 APC (clone 29F.1A12), Rat Anti-Mouse PDL1 APC (clone 10F.9G2), Rat Anti-Mouse/Human CD11b PE-Cy7 (Clone M1/70), Armenian Hamster Anti-Mouse CD11c PE-Cy7 (Clone N418), Rat Anti-Mouse CD19 PE-Cy7 (clone 6D5), Rat Anti-Mouse CD25 PE-Cy7 (clone PC61), Rat Anti-Mouse CD86 PE-Cy7 (clone GL-1), Rat Anti-Mouse ICOS PE-Cy7 (clone 7E.17G9), Rat Anti-Mouse LAG3 PE-Cy7 (clone C9B7W), Rat Anti-Mouse MHCII I-A/I-E PE-Cy7 (clone M5/114.15.2), Mouse Anti-Mouse NK1.1 PE-Cy7 (clone S17016D), Mouse Anti-Mouse TIGIT PE-Cy7 (clone 1G9), Rat Anti-Mouse CD4 AF700 (clone RM4-4), Armenian Hamster Anti-Mouse CD69 AF700 (clone H1.2F3), Armenian Hamster Anti-Mouse ICOS AF700 (clone C398.4A), Rat Anti-Mouse MHCII I-A/I-E AF700 (clone M5/114.15.2), Armenian Hamster Anti-Mouse TCRbeta AF700 (clone H57-597); From BD Biosciences: Armenian Hamster Anti-Mouse CD11c BUV395 (clone HL3), Rat Anti-Mouse CD4 BUV395 (clone GK1.5), Rat Anti-Mouse F4/80 BUV395 (clone T45-2342), Mouse Anti-Mouse TCRVbeta8.1/8.2 BUV395 (clone MR5-2), Rat Anti-Mouse CD11b BUV737 (clone M1/70), Rat Anti-Mouse CD19 BUV737 (clone 1D3), Rat Anti-Mouse CD4 BUV737 (clone GK1.5). For intracellular staining of Foxp3, the Foxp3 transcription factor staining buffer kit was used (eBioscience), using Rat Anti-Mouse FoxP3 APC (clone FJK-16s). For cell sorting, single cell suspensions from biological replicate mice were generated. Cells were sorted on a FACS ARIA FUSION cell sorter.

#### Spectral cytometry

Cells were prepared as above in Flow cytometry and cell sorting, except the cells were stained in Brilliant Stain Buffer (BD Biosciences) and analysis was performed on a Sony ID7000 spectral cytometer.

#### RNA-seq analysis

Data from GEO: GSE165817 (Elliot et al.^19^) were re-analysed using DESeq2^40^ in R version 4.0**.** Normalized read counts were transformed using the regularised log (rlog) transformation. For transcriptional profiling of *Il10*-eGFP cells, RNA was extracted from lysates using the Arcturus Picopure RNA kit (ThermoFisher) according to the manufacturer’s instructions. 5 ng of RNA was used for generation of sequencing libraries using the Quantseq 3′ mRNA-seq Library Preparation kit FWD (Lexogen). Unique Molecular Identifiers (UMIs) were used for the evaluation of input and PCR duplicates and to eliminate amplification bias. Libraries were normalised and pooled at a concentration of 4 nM for sequencing. Libraries were sequenced using the NextSeq 500 using a Mid 150v2.5 flow cell. Cluster generation and sequencing was then performed and FASTQ files generated. FASTQ files were analyzed using the BlueBee QuantSeq FWD pipeline and aligned to the GRCm38 (mm10) genome. HTSeq-count v0.6.0 was used to generate read counts for mRNA species and mapping statistics. Raw read counts in the .txt format were used for further analysis using DESeq2in R version 4.0.^40^ A DESeq dataset was created from a matrix of raw read count data. Data were filtered to remove genes with fewer than 10 reads across all samples and one biological replicate pair was removed from analysis due to being identified as an outlier using principal component analysis. Heatmap analysis was performed on the rlog transformed data using the R package gplots. Data are deposited at GEO: GSE283286.

### Quantification and statistical analysis

#### Gene set enrichment analysis

Rlog normalised counts from CD4^+^ T cells sorted 12 h post 80 ug versus 0.8 ug from Elliot et al.^19^ immunisation with [4Y]-MBP were fed into GSEA 4.3.3.^42^^,^^43^ The geneset used to test for enrichment was from Chen et al. 2021,^21^ which comprised 289 genes that were differentially expressed in human Tr1 cells versus all non-Tr1 cell subsets. Of the 249 upregulated genes from Chen et al.^21^ 119 mapped to our dataset. 1000 permutations were performed using the gene_set permutation.

#### Statistical analysis

Statistical analysis was performed on Prism 10 (GraphPad) software. For comparison of non-parametric data, a Mann Whitney U test was performed or a Kruskal Wallis test with Dunn’s multiple comparisons test. For parametric data a student t test was used. For experiments with two factors, a two-way ANOVA was performed with Sidak’s multiple comparisons testing. Variance is reported as mean ± SEM or median ± IQR for non-parametric data (unless otherwise stated); data points typically represent individual mice. ∗p = < 0.05, ∗∗p = < 0.01, ∗∗∗p = < 0.001, ∗∗∗∗p = < 0.0001.

## References

[1] Ng T.H.S., Britton G.J., Hill E.V., Verhagen J., Burton B.R., Wraith D.C. (2013). Regulation of adaptive immunity; the role of interleukin-10. Front. Immunol..

[2] Koppelman B., Neefjes J.J., De Vries J.E., De Waal Malefyt R. (1997). Interleukin-10 down-regulates MHC class II αβ peptide complexes at the plasma membrane of monocytes by affecting arrival and recycling. Immunity.

[3] D’andrea A., Aste-Amezaga M., Valiante N.M., Ma X., Kubin M., Trinchieri G. (1993). Interleukin 10 (IL-10) Inhibits human lymphocyte interferon γ-production by suppressing natural killer cell stimulatory factor/IL-12 synthesis in accessory cells. J. Exp. Med..

[4] Ito S., Ansari P., Sakatsume M., Dickensheets H., Vazquez N., Donnelly R.P., Larner A.C., Finbloom D.S. (1999). Interleukin-10 inhibits expression of both interferon α- and interferon γ-induced genes by suppressing tyrosine phosphorylation of STAT1. Blood.

[5] Yamaoka K., Otsuka T., Niiro H., Nakashima H., Tanaka Y., Nagano S., Ogami E., Niho Y., Hamasaki N., Izuhara K. (1999). Selective DNA-binding activity of interleukin-10-stimulated STAT molecules in human monocytes. J. Interferon Cytokine Res..

[6] Waal Malefyt R.D., Haanen J., Spits H., Koncarolo M.G., Te Velde A., Figdor C., Johnson K., Kastelein R., Yssel H., De Vries J.E. (1991). Interleukin 10 (il-10) and viral il-10 strongly reduce antigen-specific human t cell proliferation by diminishing the antigen-presenting capacity of monocytes via dowm'egulation of class h major histocompatibility complex expression. J. Exp. Med..

[7] de Waal Malefyt R., Abrams J., Bennett B., Figdor C.G., De Vries J.E. (1991). Interleukin 10(IL-10) inhibits cytokine synthesis by human monocytes: An autoregulatory role of IL-10 produced by monocytes. J. Exp. Med..

[8] Gabrysova L., Nicolson K.S., Streeter H.B., Verhagen J., Sabatos-Peyton C.A., Morgan D.J., Wraith D.C. (2009). Negative feedback control of the autoimmune response through antigen-induced differentiation of IL-10-secreting Th1 cells. J. Exp. Med..

[9] Roncarolo M.G., Gregori S., Bacchetta R., Battaglia M., Gagliani N. (2018). The Biology of T Regulatory Type 1 Cells and Their Therapeutic Application in Immune-Mediated Diseases. Immunity.

[10] Awasthi A., Carrier Y., Peron J.P.S., Bettelli E., Kamanaka M., Flavell R.A., Kuchroo V.K., Oukka M., Weiner H.L. (2007). A dominant function for interleukin 27 in generating interleukin 10-producing anti-inflammatory T cells. Nat. Immunol..

[11] Pot C., Jin H., Awasthi A., Liu S.M., Lai C.Y., Madan R., Sharpe A.H., Karp C.L., Miaw S.C., Ho I.C., Kuchroo V.K. (2009). Cutting edge: IL-27 induces the transcription factor c-Maf, cytokine IL-21, and the costimulatory receptor ICOS that coordinately act together to promote differentiation of IL-10-producing Tr1 cells. J. Immunol..

[12] Chen L., Flies D.B. (2013). Molecular mechanisms of T cell co-stimulation and co-inhibition. Nat. Rev. Immunol..

[13] Thaventhiran T., Sethu S., Yeang H.X.A., Al-Huseini L., Hamdam J., Sathish J.G. (2012). T cell co-inhibitory receptors-functions and signalling mechanisms. J. Clin. Cell. Immunol..

[14] Okamura T., Fujio K., Shibuya M., Sumitomo S., Shoda H., Sakaguchi S., Yamamoto K. (2009). CD4+CD25-LAG3+ regulatory T cells controlled by the transcription factor Egr-2. Proc. Natl. Acad. Sci. USA.

[15] Iwasaki Y., Fujio K., Okamura T., Yanai A., Sumitomo S., Shoda H., Tamura T., Yoshida H., Charnay P., Yamamoto K. (2013). Egr-2 transcription factor is required for Blimp-1-mediated IL-10 production in IL-27-stimulated CD4+ T cells. Eur. J. Immunol..

[16] Gabrysova L., Wraith D.C. (2010). Antigenic strength controls the generation of antigen-specific IL-10-secreting T regulatory cells. Eur. J. Immunol..

[17] Burton B.R., Britton G.J., Fang H., Verhagen J., Smithers B., Sabatos-Peyton C.A., Carney L.J., Gough J., Strobel S., Wraith D.C. (2014). Sequential transcriptional changes dictate safe and effective antigen-specific immunotherapy. Nat. Commun..

[18] Correa K., Dustin M.L. (2021). Locked and loaded: strong TCR signaling primes anti-PD-1 therapy. Trends Immunol..

[19] Elliot T.A.E., Jennings E.K., Lecky D.A.J., Thawait N., Flores-Langarica A., Copland A., Maslowski K.M., Wraith D.C., Bending D. (2021). Antigen and checkpoint receptor engagement recalibrates T cell receptor signal strength. Immunity.

[20] Jennings E., Elliot T.A.E., Thawait N., Kanabar S., Yam-Puc J.C., Ono M., Toellner K.M., Wraith D.C., Anderson G., Bending D. (2020). Nr4a1 and Nr4a3 Reporter Mice Are Differentially Sensitive to T Cell Receptor Signal Strength and Duration. Cell Rep..

[21] Chen P.P., Cepika A.M., Agarwal-Hashmi R., Saini G., Uyeda M.J., Louis D.M., Cieniewicz B., Narula M., Amaya Hernandez L.C., Harre N. (2021). Alloantigen-specific type 1 regulatory T cells suppress through CTLA-4 and PD-1 pathways and persist long-term in patients. Sci. Transl. Med..

[22] McCulloch L., Alfieri A., McColl B.W. (2018). Experimental Stroke Differentially Affects Discrete Subpopulations of Splenic Macrophages. Front. Immunol..

[23] Moran A.E., Holzapfel K.L., Xing Y., Cunningham N.R., Maltzman J.S., Punt J., Hogquist K.A. (2011). T cell receptor signal strength in Treg and iNKT cell development demonstrated by a novel fluorescent reporter mouse. J. Exp. Med..

[24] Elliot T.A.E., Jennings E.K., Lecky D.A.J., Rouvray S., Mackie G.M., Scarfe L., Sheriff L., Ono M., Maslowski K.M., Bending D. (2022). Nur77-Tempo mice reveal T cell steady state antigen recognition. Discov. Immunol..

[25] Mumm J.B., Emmerich J., Zhang X., Chan I., Wu L., Mauze S., Blaisdell S., Basham B., Dai J., Grein J. (2011). IL-10 Elicits IFNγ-Dependent tumor immune surveillance. Cancer Cell.

[26] Giovarelli M., Musiani P., Modesti A., Dellabona P., Casorati G., Allione A., Consalvo M., Cavallo F., Di Pierro F., De Giovanni C. (1995). Local release of IL-10 by transfected mouse mammary adenocarcinoma cells does not suppress but enhances antitumor reaction and elicits a strong cytotoxic lymphocyte and antibody-dependent immune memory. J. Immunol..

[27] Holsken O., Miller M., Cerwenka A. (2015). Exploiting natural killer cells for therapy of melanoma. J Dtsch Dermatol Ges.

[28] Schröder M., Meisel C., Buhl K., Profanter N., Sievert N., Volk H.D., Grütz G. (2003). Different modes of IL-10 and TGF-β to inhibit cytokine-dependent IFN-γ production: Consequences for reversal of lipopolysaccharide desensitization. J. Immunol..

[29] Bending D., Prieto Martín P., Paduraru A., Ducker C., Marzaganov E., Laviron M., Kitano S., Miyachi H., Crompton T., Ono M. (2018). A timer for analyzing temporally dynamic changes in transcription during differentiation in vivo. J. Cell Biol..

[30] Bevington S.L., Ng S.T.H., Britton G.J., Keane P., Wraith D.C., Cockerill P.N. (2020). Chromatin Priming Renders T Cell Tolerance-Associated Genes Sensitive to Activation below the Signaling Threshold for Immune Response Genes. Cell Rep..

[31] Zheng Y., Josefowicz S., Chaudhry A., Peng X.P., Forbush K., Rudensky A.Y. (2010). Role of conserved non-coding DNA elements in the Foxp3 gene in regulatory T-cell fate. Nature.

[32] Rubtsov Y.P., Rasmussen J.P., Chi E.Y., Fontenot J., Castelli L., Ye X., Treuting P., Siewe L., Roers A., Henderson W.R. (2008). Regulatory T cell-derived interleukin-10 limits inflammation at environmental interfaces. Immunity.

[33] Martinez G.J., Pereira R.M., Äijö T., Kim E.Y., Marangoni F., Pipkin M.E., Togher S., Heissmeyer V., Zhang Y.C., Crotty S. (2015). The transcription factor NFAT promotes exhaustion of activated CD8(+) T cells. Immunity.

[34] Liu J., Guan X., Ma X. (2007). Regulation of IL-27 p28 gene expression in macrophages through MyD88- and interferon-gamma-mediated pathways. J. Exp. Med..

[35] Bending D., Paduraru A., Ducker C.B., Prieto Martín P., Crompton T., Ono M. (2018). A temporally dynamic Foxp3 autoregulatory transcriptional circuit controls the effector Treg programme. EMBO J..

[36] Li M.O., Rudensky A.Y. (2016). T cell receptor signalling in the control of regulatory T cell differentiation and function. Nat. Rev. Immunol..

[37] Kamanaka M., Kim S.T., Wan Y.Y., Sutterwala F.S., Lara-Tejero M., Galán J.E., Harhaj E., Flavell R.A. (2006). Expression of interleukin-10 in intestinal lymphocytes detected by an interleukin-10 reporter knockin tiger mouse. Immunity.

[38] Hall A.O., Beiting D.P., Tato C., John B., Oldenhove G., Lombana C.G., Pritchard G.H., Silver J.S., Bouladoux N., Stumhofer J.S. (2012). The cytokines interleukin 27 and interferon-gamma promote distinct Treg cell populations required to limit infection-induced pathology. Immunity.

[39] Price A.E., Reinhardt R.L., Liang H.E., Locksley R.M. (2012). Marking and quantifying IL-17A-producing cells in vivo. PLoS One.

[40] Love M.I., Huber W., Anders S. (2014). Moderated estimation of fold change and dispersion for RNA-seq data with DESeq2. Genome Biol..

[41] Jennings E.K., Lecky D.A.J., Ono M., Bending D. (2021). Application of dual Nr4a1-GFP Nr4a3-Tocky reporter mice to study T cell receptor signaling by flow cytometry. STAR Protoc..

[42] Mootha V.K., Lindgren C.M., Eriksson K.F., Subramanian A., Sihag S., Lehar J., Puigserver P., Carlsson E., Ridderstråle M., Laurila E. (2003). PGC-1alpha-responsive genes involved in oxidative phosphorylation are coordinately downregulated in human diabetes. Nat. Genet..

[43] Subramanian A., Tamayo P., Mootha V.K., Mukherjee S., Ebert B.L., Gillette M.A., Paulovich A., Pomeroy S.L., Golub T.R., Lander E.S., Mesirov J.P. (2005). Gene set enrichment analysis: a knowledge-based approach for interpreting genome-wide expression profiles. Proc. Natl. Acad. Sci. USA.

