# Supplementary material for: Interferon-γ and IL-27 positively regulate type 1 regulatory T cell development during adaptive tolerance
Source: iScience. 2025 Mar 28;28(5):112308. doi: 10.1016/j.isci.2025.112308 (PMC12018090; doi:10.1016/j.isci.2025.112308)
Supplement: Document S1. Figures S1–S6 [file mmc1.pdf]

iScience, Volume *28*

Supplemental information

Interferon-γ and IL-27 positively regulate type 1 regulatory T cell development during adaptive tolerance

David A.J. Lecky, Lozan Sheriff, Sophie T. Rouvray, Lorna S. George, Alastair Copland, Rebecca A. Drummond, David C. Wraith, and David Bending

Supplementary Figure 1

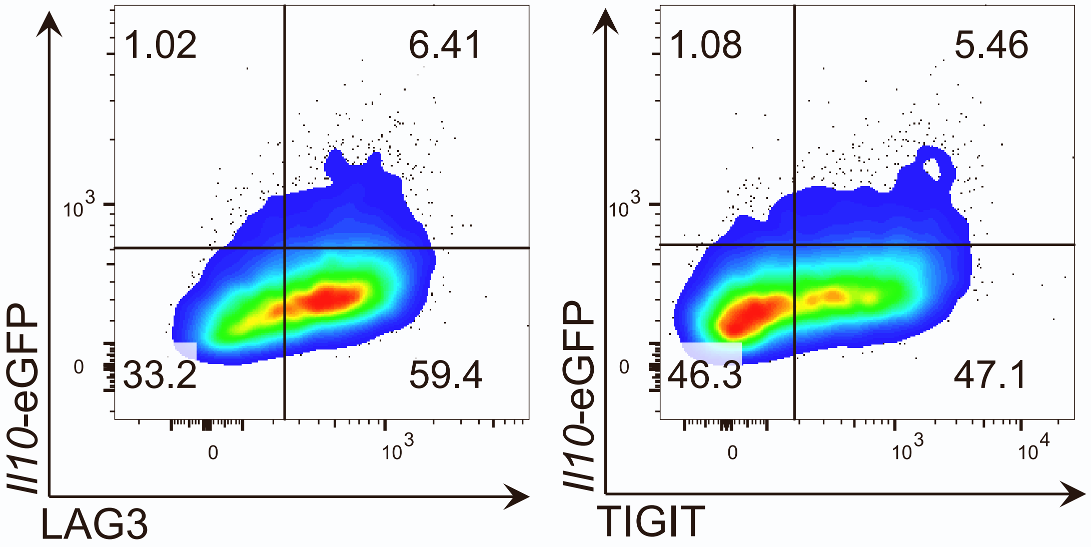

Figure S1: *Il10*-eGFP is co-expressed with Lag3 and Tigit (related to Figure 1)

Tg4 *Nr4a3*-Tocky *Il10*-eGFP were immunised with 80 µg [4Y]-MBP in PBS s.c. and splenic CD4+ T cells analysed at 24 hrs.

Supplementary Figure 2

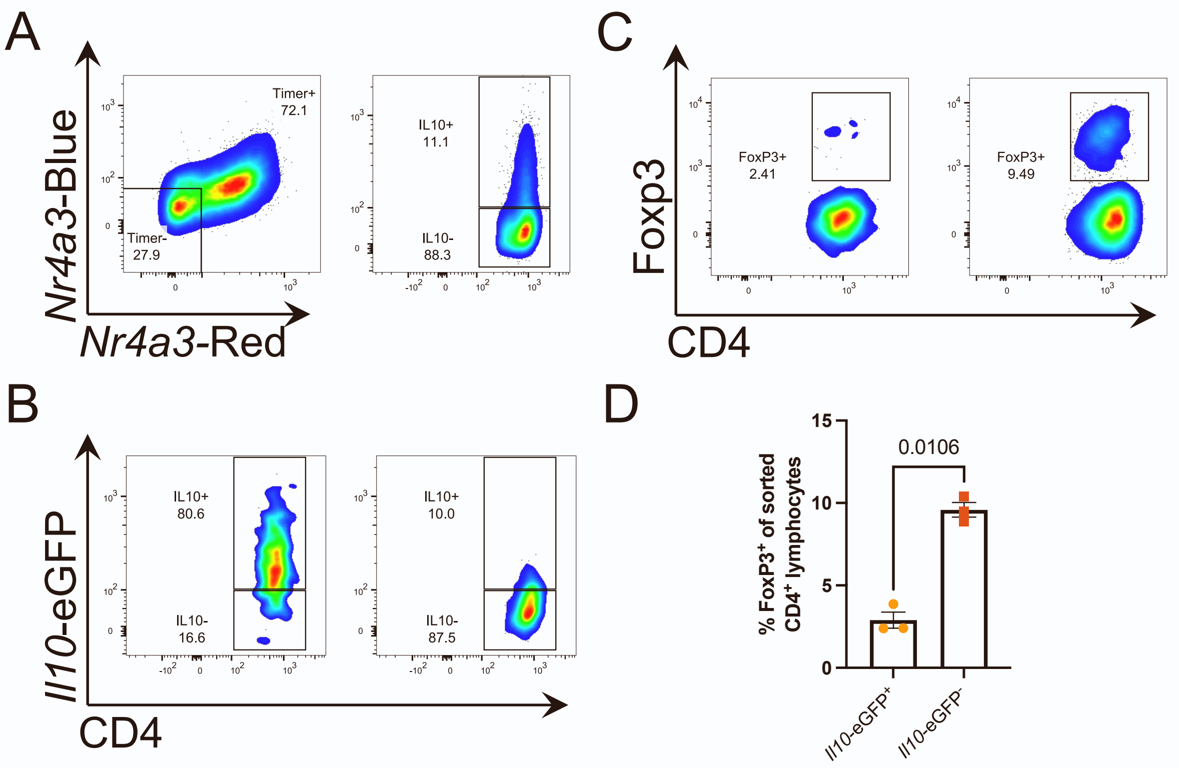

Figure S2: *Il10*-eGFP^{+} T cells do not express Foxp3 (related to Figure 1)

(**A-D**) Tg4 *Nr4a3*-Tocky *Il10*-eGFP mice were administered 4 mg/kg [4Y]-MBP in 200 µL PBS s.c. and spleens were harvested at 24 hr. Representative flow plots of CD4^{+} TCRvβ8.1/8.2^{+} pre- sort showing *Nr4a3*-Timer and *Il10*-eGFP expression **(A)**, post-sort *Il10*-eGFP in *Nr4a3*-Timer^{+} **(B)**, and post-sort FoxP3 expression in CD4^{+} TCRvβ8.1/8.2^{+} *Nr4a3*-Timer^{+} *Il10*-eGFP$^{\pm}$ **(C)**. Summary of FoxP3 frequency by *Il10*-eGFP$^{\pm}$ **(D)**. Bars represent mean+/- SEM, statistical analysis by paired t test. N = 3 per group.

Supplementary Figure 3

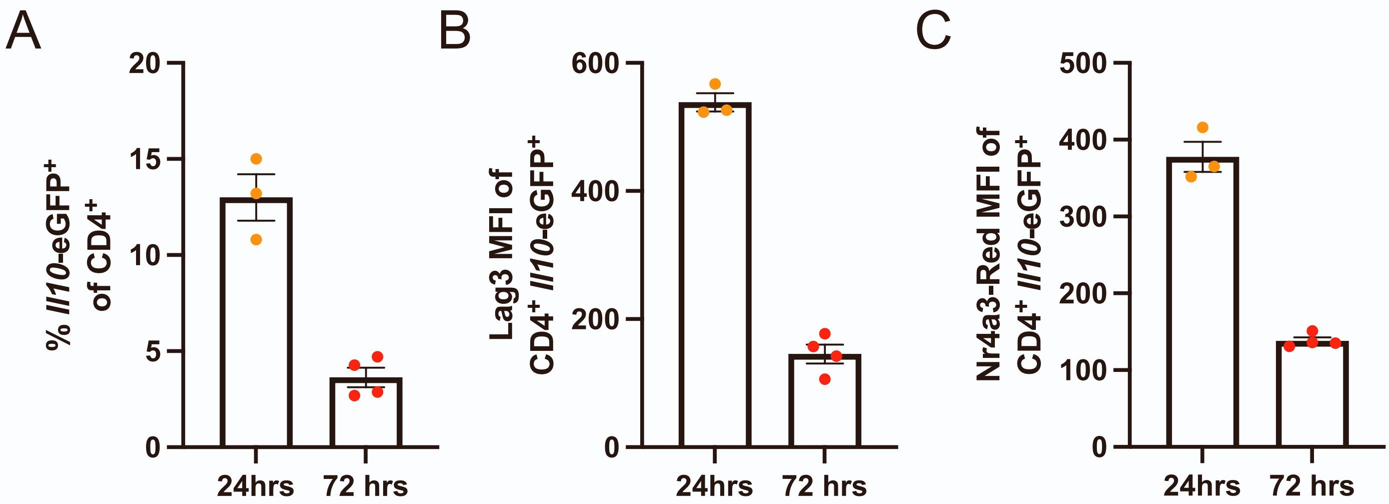

Figure S3: Transient nature of Tr1-cells following single immunisation (related to Figure 2)

Tg4 *Nr4a3*-Tocky *Il10*-eGFP were immunised with 4 mg/kg [4Y]-MBP in PBS s.c. and splenic CD4+ T cells analysed at 24 or 72 hrs. (**A**) Frequency of *Il10*-eGFP in $CD4^+$ T cells. Lag3 MFI (**B**) or Nr4a3-Red MFI (**C**) in $CD4^+$ *Il10*-eGFP T cells. N=3, bars represent mean+/-SEM.

Supplementary Figure 4

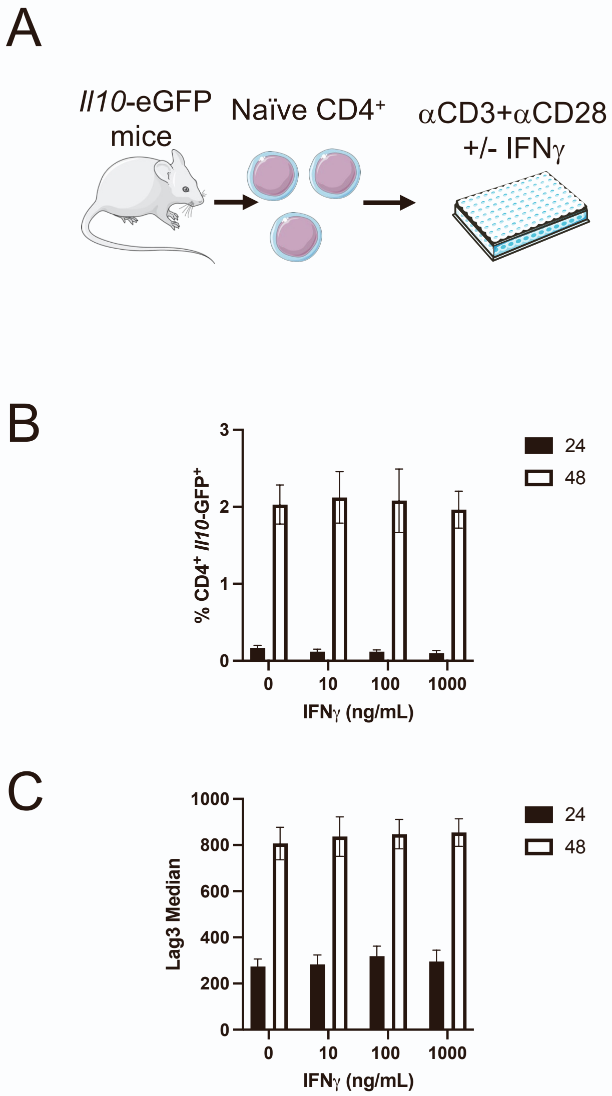

Figure S4: IFNγ has no direct effect on *Il10*-eGFP expression during T cell activation in vitro (related to Figure 3)

(**A**) Naïve CD4$^+$ T cells from *Il10*-GFP mice were isolated and cultured as indicated. 24-48 h after culture, CD4$^+$ T cell expression of (**B**) *Il10*-eGFP or (**C**) Lag3 was evaluated by flow cytometry. Bars represent mean+/-SEM.

Supplementary Figure 5

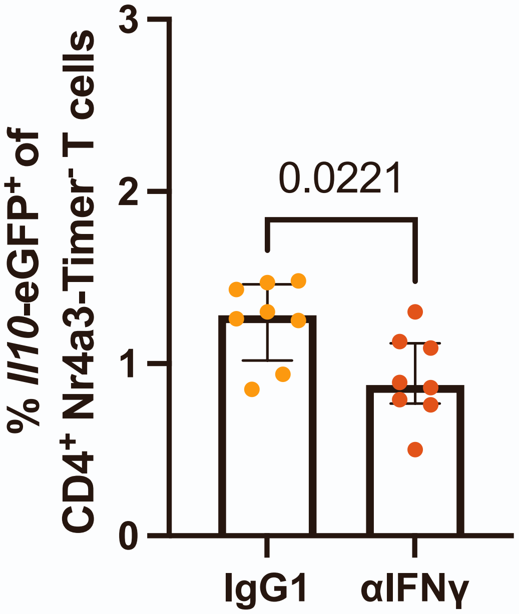

Figure S5: Changes in *Il10*-eGFP frequency in Nr4a3-Timer- T cells (related to Figure 3)

Tg4 *Nr4a3*-Tocky *Il10*-eGFP *Ifng*-YFP mice received 1 mg αIFNγ or αIgG1 isotype in 200 μL PBS i.p before immunisation with 4 mg/kg [4Y]-MBP in PBS s.c. and and spleens were harvested at 24 hr. The frequency of *Il10*-eGFP+ T cells within CD4+ Nr4a3-Timer- T cells was evaluated between the two treatment groups. N=8, bars represent median+/-IQR. Statistical analysis by Mann Whitney U Test.

Supplementary Figure 6

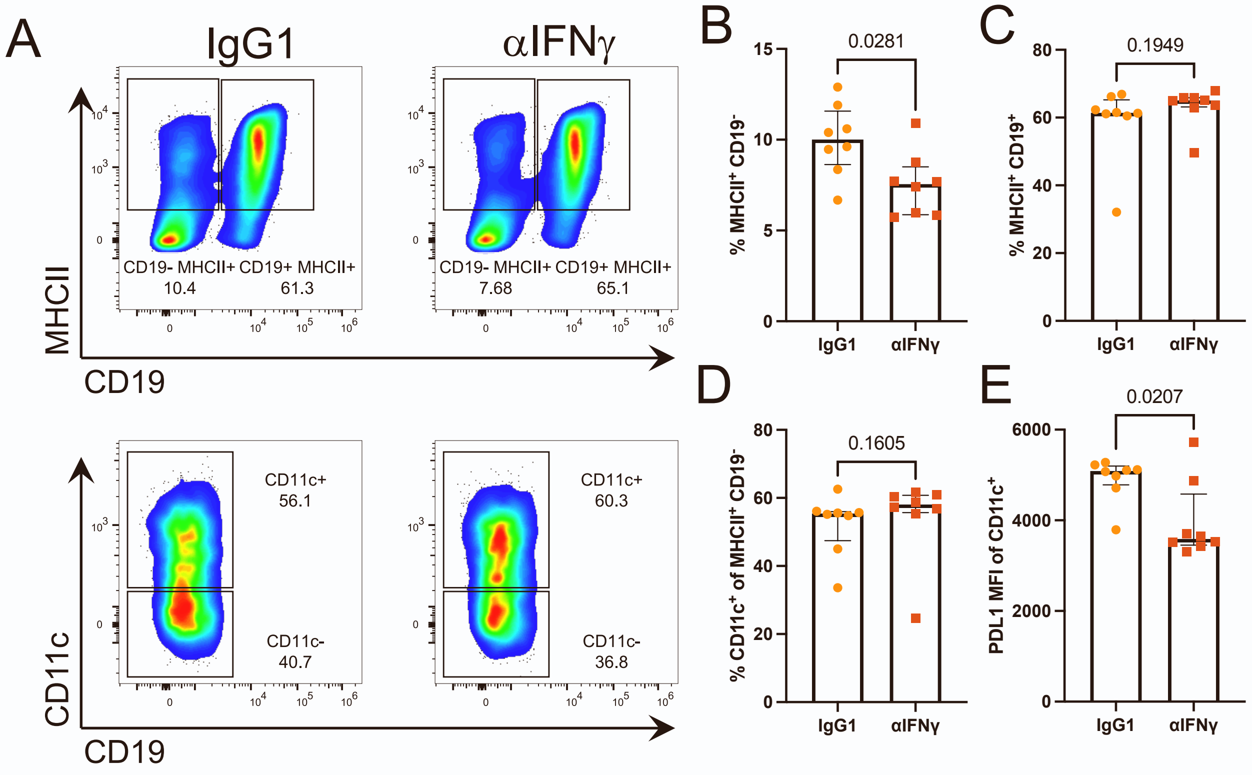

Figure S6: Effect of αIFNγ on B cell and Dendritic cell responses (related to Figure 3)

Tg4 *Nr4a3*-Tocky *Il10*-eGFP *Ifng*-YFP mice were administered 4 mg/kg [4Y]-MBP in PBS s.c. and 1 mg αIFNγ or αIgG1 isotype in 200 µL PBS i.p. and spleens were harvested. Representative flow plots at 24 hr showing MHCII against CD19 and MHCII$^+$ CD19$^-$ derived CD11c$^\pm$ under αIFNγ or αIgG1 treatment **(A)**. Summary of changes in MHCII$^+$ CD19$^-$ **(B)**, MHCII$^+$ CD19$^+$ **(C)** from **(A)**, and CD11C$^+$ **(D)** frequency from **(B)**. Summary of changes in PD-L1 MFI **(E)** from **(D)**. **(B-E)** bars represent median with interquartile range. Statistical analysis by Mann-Whitney test. N = 8 **(B-E)** per treatment.
